# Supplementary material for: The late Holocene demise of a sublittoral oyster bed in the North Sea
Source: PLoS One. 2021 Feb 16;16(2):e0242208. doi: 10.1371/journal.pone.0242208 (PMC7886217; doi:10.1371/journal.pone.0242208)
Supplement: S1 File — (PDF) [file pone.0242208.s001.pdf]

S1 File Supplementary material

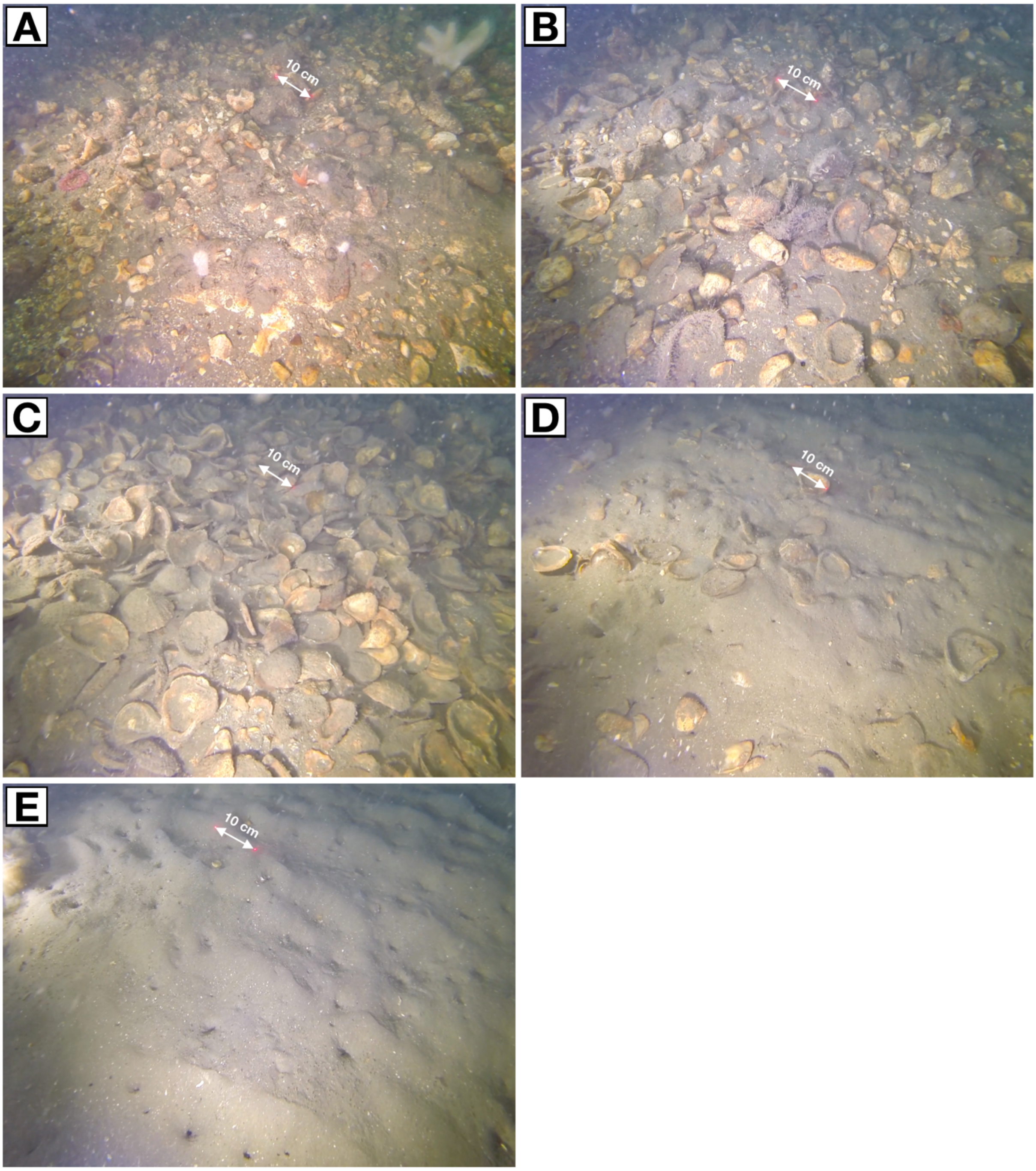

Fig S1: Examples of drift-video stills from the the five seafloor classes used in Figure 1C: (A) stones, (B) oysters and stones, (C) oysters, (D) oysters and mud, (E) mud.

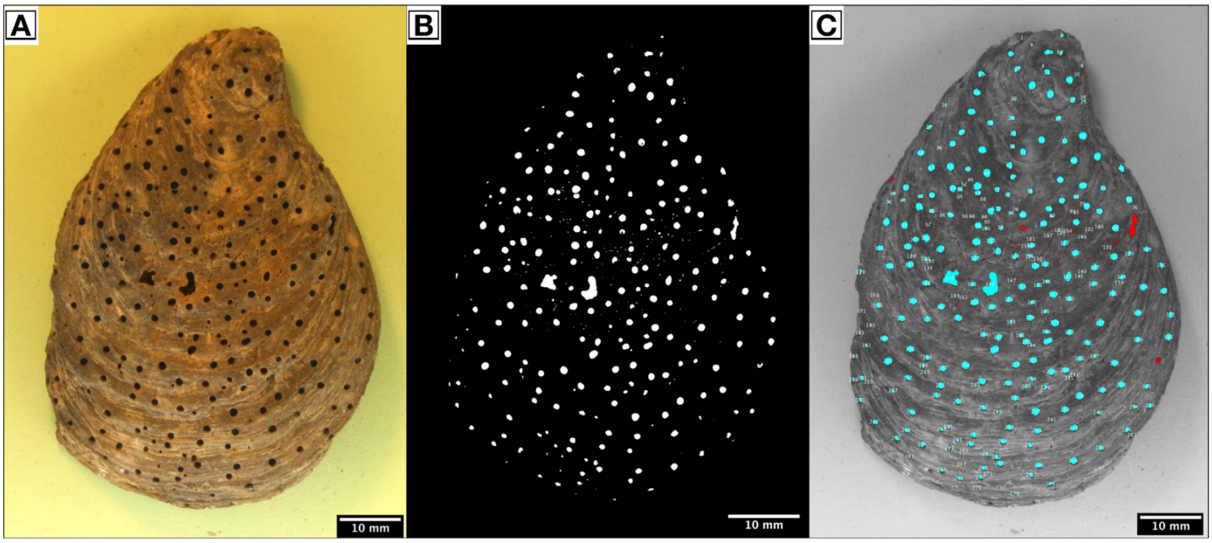

Fig S2: *Ostrea edulis* shell with boring holes of *Cliona* sp.: a) original image, b) detection of potential holes via colour and brightness thresholds with ImageJ (hue: 0-28, saturation: 0-225, brightness: 129-225, thresholding method: default, threshold color: red, color space: HSB), c) identification of hole number and area (cyan) with ImageJ. Red areas indicate potential holes that were detected but not counted. Based on biological characteristics of *Cliona* boring, the selection was set on size (area) and shape (circularity) of the holes. The highlighted potential boring holes were finally analyzed by using the "analyze particles" tool (size=0.03-10023.53 pixels, circularity=0.45-1.00), whereby too large or too small-sized and non-circular areas were rejected and not included in the count.
