# Supplementary material for: The late Holocene demise of a sublittoral oyster bed in the North Sea
Source: PLoS One. 2021 Feb 16;16(2):e0242208. doi: 10.1371/journal.pone.0242208 (PMC7886217; doi:10.1371/journal.pone.0242208)
Supplement: Attachment — Submitted filename: manuscript_ostrea_PLOSone.doc [file pone.0242208.s002.doc]

**Title (long):** A late Holocene oyster bed in the sublittoral zone of the southern North Sea: Drivers of its preindustrial demise and implications for restoration

**Title (short):** The Holocene demise of a sublittoral oyster bed in the North Sea

**Authors:** Lasse Sander^1^, H. Christian Hass^1^, Rune Michaelis^1^, Christopher Groß^2^, Tanja Hausen^2^, Bernadette Pogoda^1,2^

**Affiliations:** ^1^Alfred Wegener Institute, Helmholtz Centre for Polar and Marine Research, Wadden Sea Research Station, List/Sylt; ^2^Alfred Wegener Institute, Helmholtz Centre for Polar and Marine Research, Shelf Sea System Ecology, Bremerhaven

**Corresponding author:** Lasse Sander, Alfred Wegener Institute, Helmholtz Centre for Polar and Marine Research, Wadden Sea Research Station, Hafenstraße 43, 25992 List/Sylt, Germany, email: lasse.sander@awi.de, tel.: +49 4651/8564117, ORCID: 0000-0003-1066-7952

**Abstract:** A fossil oyster bed (*Ostrea edulis*) was recently encountered offshore Helgoland (German Bight). The European flat oyster *Ostrea edulis* has been a keystone species in the North Sea, but declined massively in the early 20th century. The ecological restoration of *Ostrea* habitats is a current focal point in the entire North Sea area. In order to better understand the mechanisms that caused the local collapse of the oyster population, this study investigates the size structure, weight, and age of the shells, along with the spatial dimensions, seafloor properties, and environmental context of the oyster bed. The results show that the demise of the population occurred around 700 CE, ruling out excessive harvest as a driver of decline. Synchronicity of increased geomorphological activity of rivers and concurrent major land use changes in early medieval Europe suggest that increased sedimentation may have been a viable stressor to explain a reduced performance of the oysters. The shells provide no indication of a demographically poor state of the oyster bed prior to its demise, but bear evidence of the wide-spread occurrence of the boring sponge *Cliona sp*. Our study challenges the assumption of a stable preindustrial state of the North Sea, and we conclude that the long-term variability of environmental conditions needs to be addressed in order to benchmark success criteria for the conservation and restoration of marine species and habitats.

**1. Introduction**

The European flat oyster (*Ostrea edulis*) is a marine filter-feeding bivalve native to the larger North Sea area and historically occurred in vast populations occupying sublittoral environments across different habitats [1–4]. *Ostrea edulis* beds are characterized by a high structural complexity and provide shelter and settling ground for a range of associated species, thus increasing local biodiversity [5–7]. Oysters further hold both cultural and commercial value, and provide a range of ecosystem services positively affecting the overall quality of marine and brackish environments [3, 8, 9]. Unfortunately, oyster populations have declined throughout Europe in recent historical times, which is attributed to the massive extraction and disturbance by targeted and non-targeted fisheries from the beginning of the industrial period [10–13]. In the mid-20^th^ century, *O. edulis* became functionally extinct in the southern North Sea (i.e. >99% lost) [14–16]. Several national and international projects are currently working towards the reestablishment and restocking of *O*. *edulis* in European waters [9]. Small numbers of flat oysters are occasionally found in the North Sea, suggesting a restorative potential of the species in the area. However, it remains unclear, which environmental parameters have supported the development of a large population of *O*. *edulis* in offshore environments in the past [8, 17–19].

Despite the interest in restoration, our current knowledge on the past distribution and ecological state of oyster stocks remains limited and largely conceptual [1]; A situation that grants interpretative leeway in the assessment of changes in the state of populations and habitats. Historical harvest statistics document the massive extraction of marketable specimen, and past abundance is often taken as proxy for the healthy state of oyster populations, a view that is further supported by the wide spatial distribution of *O*. *edulis* indicated in historical maps [12–15, 20, 21].

In the German Bight, a general distinction between the historical *Ostrea* beds of the Wadden Sea and the so-called offshore or deep-water populations (also referred to as “Helgoländer” [22]) has to be made. The environmental conditions in both areas are significantly different (e.g. water depth, hydrodynamics), and the intensity of exploitation varied largely. The amounts of oysters extracted from the Wadden Sea have been more than an order of magnitude higher than those harvested from the open North Sea and targeted near-shore fishery has been maintained considerably longer [15, 23]. In the late 18^th^ century, large amounts of oysters were found in proximity to the offshore island Heligoland but were not commonly fished due to the lack of adequate equipment [24, 25]. In the open North Sea, oyster beds probably remained unknown until their discovery in the mid-19^th^ century, which was followed by a massive increase in the intensity of use [15, 26]. Until the 1870s, overexploitation had already decreased landings significantly and problems with recruitment had become evident [27]. In the year 1900, the oyster beds around Helgoland have been described as disproportionally old, characterized by the lack of juveniles and more than half of the population as being infested by the boring sponge *Cliona celata* [27]. Harvest was discontinued at the beginning of World War I, but oysters did not recover [27].

Ecological restoration of European flat oysters and its habitat is expected to result in the provision of a range of desirable ecosystem functions and services for the southern North Sea. These functions include an increased biodiversity and habitat complexity, which should result in the provision of food, shelter, spawning ground and settlement substrate, along with the local enhancement of benthopelagic-coupling and water quality [28]. Accordingly, in the context of marine nature conservation, the ecological role of biogenic reefs and ecosystem engineers is valued superior. A number of trials are currently initiated in the southern North Sea [9, 16, 29]. The success is, however, contingent upon the existence of suitable habitat conditions that allow the development of a stable oyster population. Due to the substantial loss of oyster beds in the German Bight and the severely degraded state of the remaining populations, no profound ecological baseline and no reliable data on former biogenic reef structures in the sublittoral zone exist.

This study investigates a recently discovered fossil oyster bed located offshore Helgoland in order to facilitate a long missing description of a deep-sublittoral oyster bed in the North Sea. The aims of this study are (1) to obtain basic information on the location, composition, and age structure of the oyster bed, (2) to provide a new perspective on the past existence and state of oysters located within the eastern expansion of the historical “oyster grounds”, and (3) to discuss the long-term variability of potential environmental stressors (other than direct human impact) as a benchmark to inform current oyster restoration initiatives.

**2. Methods**

*2.1 Study area*

The object of this study is an oyster bed that was found 3.3 km west of Helgoland during a drift-video survey in summer 2017 [30]. The island of Heligoland is located in the German Bight (south-eastern North Sea) at a distance of approx. 40–45 km offshore from the East and North Frisian Wadden Sea (cf. Fig 1A). Salinities in the waters around Helgoland range around 30–32 [31, 32] and the area is influenced by the discharge of freshwater from rivers (Ems, Weser, Elbe) [33, 34]. The south-eastern North Sea receives inflow of Atlantic waters, primarily through the English Channel, and currents from western and south-western directions dominate [35–38]. Tide-induced surface currents can reach velocities of up to >0.6 m/sec during spring tides [39]. The seafloor around Helgoland is gently inclined and diverse in its geological composition, including bedrock and rubble composed of Triassic sandstone, Cretaceous limestone with flint, and Holocene sands and mud, which is also reflected in the bathymetry as well as in the presence of different bedrock, hard substrates and sediment types [40, 41]. This situation determines the availability of a range of different habitat conditions for sessile organisms, such as water depth, current exposure, inclination, and sediment composition, which define light penetration, phytoplankton concentration and food availability, as well as larval distribution. The fossil oyster bed is located entirely within the nature conservation area “Helgoländer Felssockel” (Fig 1B).

**Fig 1:** (A) Overview map of the North Sea basin with the historical distribution of *Ostrea edulis* [20], (B) Bathymetry and location of the studied oyster bed offshore Helgoland, (C) Drift-video transects and the surface composition around the oyster bed, (D) Still-image example of oyster shells under a thin veneer of sediment (NB: laser for scale = 10 cm). See Fig S1 for examples of all seafloor classes.

*2.2 Field data*

2.2.1 Drift video

A ship-based survey of the fossil oyster bed was conducted on board RV “Mya II” between June 17 – 20, 2019. Video data was obtained using two ahead-oriented cameras (CT3009, C-Tecnics, Aberdeen, Scotland; resolution: 1920 x 1072, 30 frames per second; GoPro, HERO 3+ black edition, GoPro, Inc., San Mateo, California; resolution: 1920 x 1440, 47.95 frames per second) mounted to a metal frame. To improve ambient light conditions, four LED-lamps (CT4004, C-Tecnics, Aberdeen Scotland) were attached to the frame along with two underwater lasers at a reference spacing of 10 cm. The video frame was deployed by a metal wire from the drifting ship (velocity < 1 knot) using a small crane. The camera frame was kept close to the sea floor and repeatedly lowered to the ground in order to improve the quality of the video material.

2.2.2 Grab sampling

Locations for grab sampling were selected based on the video data and chosen to represent the different densities/compositions of the oyster bed, and variation along and across the bed. Sampling was conducted using a HELCOM grab sampler with a volume of 40 liters. The contents of the grab were transferred into a plastic container. All oyster shells were picked and rinsed with sea water, including fractured specimen allowing an estimation of the original shell size and properties (i.e. preservation of the umbo and >50% of the estimated shell area). All other fragments were discarded.

2.2.3 Background data

The interpretation of the field data is supported by a digital bathymetric model for the German Bight (BSH 2018) and additional data from AufMod [43] and GEBCO gridded bathymetric data [44]. Modelled current velocities for selected locations within the oyster bed were provided by the Operational Circulation Model (BSHcmod) [45].

*2.3. Sample analysis*

In the laboratory, the samples were dried at 48°C for 72 hours. All specimen from all samples were cleaned with a brush and weighed on a laboratory scale (in grams with an accuracy of two decimals). Samples were photographed under artificial light (Canon EOS 7D Mark II with Canon EF-S 18–55mm *f*/3.5–5.6 lens) from both sides (interior, exterior) and were measured (length, width) using ImageJ 1.52q [46]. The projected 2D oyster area was determined by highlighting the shell, using the “color threshold” tool (hue: 0–28, saturation: 0–243, brightness: 0–230, thresholding method: default, threshold color: red, color space: HSB), and a subsequent measurement of the oyster area by the “wand tracing” tool. To assess the number and area of boring sponge holes for each individual oyster, the original image was modified in color (converted to 8-bit, colorized with “spectrum”, converted to RGB) and consecutively the identified holes were highlighted by color thresholding (see Fig S2).

*2.4 Radiocarbon dating*

Specimen of *O*. *edulis* from each grab location were selected for age determination based on size, and state of preservation. A total number of 19 samples were used for radiocarbon dating, composed of one large shell from each grab location and three additional juvenile shells from three locations selected based on spatial distribution. All material was obtained from the outermost (youngest) part of the respective valve. An overview of all samples is given in Table 2. The sampling areas were mechanically cleaned from overgrowth and sampled with pliers. The material was dated at the MICADAS laboratory at AWI Bremerhaven and all samples were calibrated using OxCal 4.3 [47] and the Marine 13 calibration curve [48]. Due to the unknown temporal variability of the reservoir age in the southern North Sea, the global average of 400 years was assumed to apply and no additional corrections (∆R) were used in the calibration process [49]. All ages are converted to the BCE/CE (Before the Common Era/Common Era) system to allow for consistency and easier reference with the historical data.

**3. Results**

*3.1 Location and surface properties*

The fossil oyster bed is located in water depths between 33–44 m below NN (local ordinance datum) and has a maximum length of 3000 m and a width of 400 m. It is located at a marked break in slope determined by the transition from the bedrock base of the island of Helgoland to the deeper and relatively flat North Sea shelf (Fig 1B). The oyster bed is orientated along its length axis from NNW to SSE. The seafloor at the oyster bed is inclined at an angle of c. 1°, while the slope is in similar water depths much less pronounced north of the oyster bed (c. 0.2°), but remains steep south-east of the bed (see Fig 1C). The encountered oyster shells thus form a slim band roughly aligned with the isobaths. Towards the lower (i.e. deeper) boundary of the bed, the oysters gradually dip under unconsolidated cohesive sediment, while the upper (i.e. shallower) boundary is characterized by a gradual transition towards hard substrates, with the presence of gravel- to boulder-sized material.

During the analysis of the video material the following species/groups were observed: (A) benthic sessile macrofauna: *Pomatoceros triqueter* (Polychaet), *Alcyonium digitatum* (dead man’s fingers), *Metridium senile* (frilled anemone) and other Actiniaria (sea anemones), *Flustra foliacea* and other Bryozoa/Hydrozoa spp.; (B) benthic mobile macrofauna: *Cancer pagurus* (edible crab), *Homarus gammaraus* (European lobster), *Pagurus bernhardus* (hermit crab), *Necora puber* (velvet crab), *Hyas araneus (spider crab),* Portunidae spp. (swimming crabs), *Echinus esculentus* (common sea urchin), *Asteria rubens* (common starfish), (C) benthopelagic macrofauna: Caridae spp. (shrimp), *Ctenolabrus rupestris (Goldsinny wrasse), Pleuronectes platessa (European plaice), Callionymus lyra (Common dragonet), Pholis gunnellus (rock gunnel), Agonus cataphractus,* other Cottidae, and (D) pelagic macrofauna: *Loligo vulgaris* (European squid).

*3.2 Composition of the oyster shells*

All shells from the grab samples are disarticulated and none of the valves are aggregated. However, there is a large variation of shell morphologies represented, suggesting attachment to either shell material or hard substrates. The maximum shell length is 11.0 cm (mean: 5.5 cm), and the maximum width is 9.5 cm (mean: 4.6 cm; Fig 2). This means that an estimated amount of >60% of the oysters reached maturity [50]. All size classes are present in the samples and both length and width show a bimodal distribution, with peaks in the length bins 3.0–3.5 cm and 6.5–7.0 cm, as well as in the width bins 2.0–2.5 cm and 6.5–7.0 cm. The shell weight lies between <1 g to 125 g (mean: 19 g). About 34% of the samples lie in the weight bin 0–5 g and 54% weigh between 5 and 35 g.

**Fig 2:** Overview of the length, width and weight of all measured shells (n=589) from the 16 grab sampling locations.

*3.3 Boring epifauna*

Ample traces of boring epifauna were observed on the oyster shells, mostly from the boring sponge *Cliona* sp., which can be identified by borehole size and morphology [51]. Depending on the respective stations, only 4–29% of sampled shells were not infested (see Table 1), with no preferences for right or left valves. Infestation was evenly distributed over the complete shell area (see. Fig 3), only in smaller size classes, boring predominates in the areas around the umbo. Number of holes per cm^2^ and percentage of perforated area increased with size class and reach a maximum in oyster shells with 9–10 cm length (see Fig 4). Some shells show defense structures in the hypostracum layer evidencing that boring sponges have impacted living oysters [51]. There is no relation between *Cliona* sp. prevalence and the depth or location of the grab samples.

**Table 1:** Number and percentage of non-infested oyster shells per station.

| Station | Total number of shells per station | Number of  not-infested shells | Percentage (%) of not-infested shells |
| --- | --- | --- | --- |
| 5 | 120 | 19 | 15.83 |
| 6 | 86 | 6 | 6.98 |
| 7 | 106 | 4 | 3.77 |
| 9 | 36 | 3 | 8.33 |
| 10 | 66 | 4 | 6.06 |
| 12 | 28 | 8 | 28.57 |
| 13 | 66 | 6 | 9.09 |
| 14 | 100 | 8 | 8.00 |
| 15 | 60 | 8 | 13.33 |
| 16 | 52 | 3 | 5.77 |
| 17 | 48 | 4 | 8.33 |
| 18 | 30 | 3 | 10.00 |
| 19 | 54 | 8 | 14.81 |
| 20 | 94 | 6 | 6.38 |
| 21 | 42 | 5 | 11.9 |
| 22 | 78 | 5 | 6.41 |
| 24 | 20 | 1 | 5.00 |

**Fig 3:** *Ostrea edulis* shells with boring holes of *Cliona* sp. showing different degrees of infestation: (A) adult shell, size class ≤9cm with heavy infestation covering the complete shell, (B) and (C) outer and inner side of a smaller size class with infestation around the umbo, a typical sign for ante-mortem infection, (D) and (E) outer and inner side of an un-infested smaller size class. Scale = 1 cm.

**Fig 4:** Size classes of sampled *Ostrea edulis* shells and infestation with *Cliona* sp. distributed over different size classes (length) indicated as number of holes per area and individual.

*3.4 Absolute age control*

Almost all samples (n=18) yielded calibrated radiocarbon ages between 2100 BCE – 700 CE (Table 2, Fig. 5). One sample (a juvenile oyster; Sample 12J) yielded a median age of 220 years (1800 CE). There is no apparent relationship between the depth or location of the sample with the determined radiocarbon age. From three locations, both an adult and a juvenile specimen were dated. Sample 12A and 12J, both have a very divergent age with an offset of 3,500 years. For samples 18A and 18J, the age difference is at least 1170 years, while samples 15A and 15J have almost entirely overlapping age ranges (Table 2). Hence, there appears to be no relationship between the size (i.e. relative age) of the shells and their absolute age. The dated samples are interpreted as 19 independent replicas from an undisturbed and continuous seafloor feature and are therefore deemed to represent indication of the longevity of the oyster population at the investigated site. Thereby, the ages of the shells are interpreted as minimum ages for the existence of the bed and older shells are expected to exist at lower stratigraphic positions.

**Table 2:** Overview of radiocarbon samples

| Sample ID | Lab ID | Sample bulk weight (mg) | Sample weight (µg C) | 14C age | Calibrated age range (2σ)* | % |
| --- | --- | --- | --- | --- | --- | --- |
| 12J | 3027.1.1 | 82 | 1023 | 512±22 | 1700 CE – 1810 CE | 91.1 |
| 20/1-1 | 4399.1.1 | 272 | 979 | 1830±23 | 490 CE – 660 CE | 95.4 |
| 18J | 3031.1.1 | 38 | 1017 | 1900±23 | 430 CE – 580 CE | 95.4 |
| 19/1-1 | 4398.1.1 | 101 | 994 | 2194±23 | 90 CE – 240 CE | 95.4 |
| 2/1-6 | 4401.1.1 | 89 | 893 | 2369±23 | 140 BCE – 40 CE | 95.4 |
| 5/1-2 | 4389.1.2 | 41 | 963 | 2504±24 | 340 BCE – 140 BCE | 95.4 |
| 6/1-1 | 4390.1.2 | 121 | 966 | 2513±23 | 340 BCE – 160 BCE | 95.4 |
| 9/1-7 | 4392.1.1 | 104 | 988 | 2629±23 | 450 BCE – 300 BCE | 93.1 |
| 21/1-2 | 4400.1.1 | 126 | 921 | 2684±23 | 510 BCE – 360 BCE | 95.4 |
| 15A | 3028.1.1 | 77 | 1019 | 2698±23 | 530 BCE – 370 BCE | 95.4 |
| 15J | 3029.1.1 | 56 | 1020 | 2762±23 | 660 BCE – 410 BCE | 94.1 |
| 17/1-10 | 4397.1.1 | 378 | 933 | 2781±23 | 710 BCE – 450 BCE | 95.4 |
| 7/1-8 | 4391.1.1 | 134 | 994 | 2823±23 | 740 BCE – 530 BCE | 95.4 |
| 16/1-11 | 4396.1.1 | 134 | 736 | 2911±60 | 860 BCE – 530 BCE | 95.4 |
| 18A | 3030.1.1 | 132 | 983 | 2961±23 | 840 BCE – 740 BCE | 95.4 |
| 14/2-1 | 4395.1.1 | 107 | 983 | 3205±23 | 1170 BCE – 970 BCE | 95.4 |
| 12A | 3026.1.1 | 41 | 1035 | 3378±23 | 1380 BCE – 1210 BCE | 95.4 |
| 13/1-7 | 4394.1.1 | 235 | 908 | 3838±24 | 1930 BCE – 1730 BCE | 95.4 |
| 10/1-3 | 4393.1.1 | 273 | 748 | 3937±60 | 2160 BCE – 1800 BCE | 95.4 |

* in BCE/CE, rounded to the nearest 10, calibrated using the Marine13 calibration curve (Reimer et al. 2013)

**4. Discussion**

*4.1 The past ecological state of the oyster bed*

The size structure of the material obtained suggests that the oyster bed was in a good reproductive state, given that all size classes are represented and that a large amount of specimen had apparently reached reproductive age (>60%) [50, 52–54]. The presence of small shells (down to spat size) suggests a renewal of the stock with young material from the own population or supplied from nearby, updrift locations. The presence of large specimens further indicates that enough settling substrate for spat and juvenile oysters had been present. Nevertheless, the investigated oyster bed does not represent a stratified record because we cannot make any relative age inferences between the samples obtained in one grab sample. As the age of all shells has not been determined, the size and weight of the oyster shells is interpreted as a mixed record, presenting the time integrated product of the oyster bed’s productivity over the entire period of its existence. The inferences made below do thus do not represent time slices, but reflect an overall assessment of the populations structure and indication of its “health”.

Among other stressors, boring epifauna may have affected the health of the oyster population. In the North Sea, the boring sponge *Cliona celata* is known to infest living and non-living calcium carbonate substrates, such as limestone and bivalve shells of *O*. *edulis, Crassostrea gigas* and *Crepidula fornicata*. Living oysters are infested from a certain age and size, e.g. >3 years [55] as the sponge prefers rough and weathered crevices, which are scarce on younger smooth shell surfaces [51, 56]. The infection begins in the oldest part of the oyster around the umbo and protoconch [57]. In living oysters, the left valve is more affected by the sponge, eventually due to the infection by the larvae, which approach from the substrate below. Only if food availability and energy uptake are high during spring and summer, infested oysters can counteract the boring by building up hypostracum layers [55]. Postmortem, the complete shell is infested and boreholes are uniformly distributed [51]. If investigated oyster shells have been infested ante mortem, the boring sponge posed a certain risk, assuming additional stressors came into effect. In that case, coping with the disease via respective shell building mechanisms is reduced, which significantly decreases growth and condition and eventually results in elevated mortalities [58]. Several sampled and investigated oysters have been infested ante mortem, as they show signs of secondary shell building mechanisms.

*4.2 Causes of the late Holocene demise of the oyster bed*

A number of 18 samples yielded ages older than 700 CE, with eleven samples determined to calibrated ages between 900 BCE and 100 CE. Over the entire period from 1,400 BCE to 700 CE (2,100 years) the samples show an (almost) continuous record of overlapping probabilities, suggesting the sustained presence of oysters at the studied location. A hiatus in the presence of oyster shells suggest the demise of the population prior to 700 CE, entailing the collapse and disappearance of a living oyster bed at that particular location (Fig 5).

Long-term fluctuations in the health and density of oyster populations have been linked to sedimentation as an important environmental stressor for filter-feeding organisms [e.g. 8, 10, 14, 15, 59, 60]. The data do not provide a lower limit for the establishment of the oyster bed, but appropriate habitat conditions have probably been established around Helgoland c. 8000 years ago, when the postglacial sea-level rise transgressed the southern North Sea [61]. Radiocarbon ages from Limfjord support that *O*. *edulis* has been present in the North Sea area since about that time [62, 63]. It is thus hypothesized that older shell material at our study site could be obtained from lower (buried) beds and suggest that the assemblage tapers out, which should be regarded as a sampling artefact rather than indication for the absence of oysters prior to 2000 BCE. To our surprise, the age data suggest a demise of the oyster bank prior to the documented decline of oyster stocks, ruling out the destruction of the bed by overfishing in historical times as a cause. All (but one) dated oysters are older than ca. 650 CE, and a single sample yielded a recent age falling into the age range for the known existence of oyster populations in the area. There is hence no evidence for the existence of an oyster bed between after 650 CE and historical documentation (19^th^ century).

A study of sediment cores from the so-called Helgoland Mud Area, located southeast of the island, provides evidence for very high sedimentation rates of >13 mm/yr between 350–1200 CE [64]. After 1200 CE, sedimentation decreased to 1.6–2.6 mm/yr (Fig 5A) [64]. Sedimentation rates in the mud area have previously been estimated to 1.6 mm/yr as a Holocene average [65, 66]. It thus appears that the period of high sedimentation presents a marked deviation from the Holocene average, while the inferred rates for the period 1200 CE until present are in a similar order of magnitude as the Holocene background value. A potential explanation for the occurrence of a period of increased sedimentation rates may be found in major land-use changes on the European mainland [67]. Modelled results on the timing and extent of deforestation in Europe and show that the conversion of forest cover to meadows and arable land proceeded rapidly and was at an advanced state already in the middle of the first millennium CE [67]. The timing is further consistent with a period of increased river activity in Europe starting at around 450 CE [68]. Anthropogenic land-use change may have led to a more direct coupling of land areas with riverine environment [69] and an increase in turbidity and sedimentation in the south-eastern North Sea from fluvial sources thus presents itself as a possible scenario (Fig 5C). In addition, geoarchaeological evidence suggests that a series of extreme river flood events occurred in the Elbe River after 550 CE [70]. Elbe river plume fronts commonly reach the area around Helgoland [71]. The intensified occurrence of storms may additionally have increased sedimentation in the North Sea (i.e. sediment input from storm-induced coastal erosion) in periods of thermal transition in the late Holocene (i.e. warm to cold/cold to warm periods) [72]. Based on the temporal fit between the hiatus in the oyster bed and the above-described conceptual relations, it is argued that increased sedimentation may be a viable candidate for explaining the deterioration of the habitat quality and the subsequent demise of oyster population in the North Sea. Increased sedimentation results in ineffective filtration with a decrease in the quality and quantity of food uptake and a reduced energy budget, which affects all size classes of a filter-feeding population, and furthermore, the associated filter-feeding fauna.

Similar dynamics have been proposed to explain the decline of the eastern oyster (*Crassostrea virginica*) in estuaries on the east coast of the USA. Data from the Hudson River indicates that the absence of oysters in the late Holocene can be explained by increased estuarine sedimentation that reflects the heightened influx of terrestrial sediment during that time [73]. The material originates from erosion, induced by land-cover change and its delivery has been associated with wetter climate and the possible effects of rising sea level. In Chesapeake Bay, oyster populations declined and reef complexes shrank nearly 200 years ago under the pressure of increased oyster extraction and disease, but sedimentation has contributed to the decline as an additional stressor [59]. It has further been hypothesized that a decline in oyster consumption in Denmark around 4000 BCE, as reconstructed from kitchen middens, may be attributed to a decline in oyster abundance caused by increased sedimentation from coastal erosion [74].

Even though studies demonstrate the negative impact of increased sedimentation on the health of oyster beds, other possible causes for a population decline should not be categorically excluded, such as a lack of larval supply, climate and current-pattern feedbacks, diseases (e.g. virus infections), or massive predation.

**Fig 5:** Age ranges and the summed probability distributions for the dated oysters from our study site combined with (A) past sedimentation in the Helgoland Mud Area (HMA) [64], (B) Storminess records from Atlantic Europe [72 and references therein], and (C) a record of Holocene river activity [68]. (D) The green area to the right marks the time window from the first documentation of offshore oysters in the German Bight to their functional extinction [15].

*4.3 Implications for restoration*

Our data describe the dimension of a long-standing sublittoral oyster bed with respect to specific and combined environmental parameters. They indicate that seabed characteristics (e.g. slope angle, direction of slope) define habitat conditions (e.g. current direction, bed shear stress, sedimentation) for site-specific oyster distribution. The integration of these identified factors with oyster larvae behavior [75] and into suitability index models [76] will inform site selection and define connectivity of restoration sites. The data furthermore suggest that a good state of the historical oyster populations in the German Bight prior to the dawn of industrial fisheries in the late 19^th^/early 20^th^ century may stand less clear [e.g. 4, 15, 23] and that ideal habitat conditions for the restoration of *O*. *edulis* cannot be inferred from the historical/early industrial state of the North Sea. Early anthropogenic stressors, such as increased sedimentation from terrestrial erosion may be a factor that has previously been underestimated. Spatial shifts in the ecological quality of the environment, which can occur on shorter or larger time scales, drive the extension and health of oyster populations with regard to sedimentation rates and food availability. This stresses the importance of holistic approaches to inform the restoration and conservation of marine habitats. More specifically, the site selection for restoration measures needs to consider the potential validity of sedimentation rates as crucial factors [19]. Furthermore, studies should address the variability of environmental stressors that may jeopardize restorative efforts, as these present additional parameters adding to problems of pure oyster biology and ecology.

**5. Conclusions**

The study of a fossil oyster bed in the southern North Sea showed that the native oyster (*O*. *edulis*) successfully lived for at least 2,500 years at a location where no living specimen are found today. The dimension and extension of the fossil oyster bed suggest that self-sustaining recruitment was possible and that environmental parameters limited the extent of the bed. The demise, however, appears to have happened more than 1300 years ago and the absence of living oysters cannot be explained by intensive fisheries and overexploitation. We propose that the local population went through a phase of deteriorating habitat conditions, primarily controlled by intensified sedimentation, which ultimately lead to the collapse of the oyster bed. This case study challenges the general assumption that the natural habitats of the North Sea have been in a pristine or optimal state prior to the industrial period. Early anthropogenic land-use changes may have had a more considerable negative effect on the performance of benthic filter-feeders than previously assumed, and the past period of success of *O*. *edulis* in the North Sea may have happened under more favorable boundary conditions. These results imply that habitat deterioration by sedimentation may impede ecosystem recovery and that robust assessments of past ecological states can provide relevant baseline information for ecological restoration. The restoration efforts of *O*. *edulis* and *Ostrea* habitats thus need to consider the long-term variability of environmental conditions and past ecological states in order to achieve lasting ecological benefits.

**Acknowledgements**

The captain and crew of the research vessels FK “Uthörn” and FS “Mya II” are thanked for their collaboration and commitment during the field work. Gesine Mollenhauer and Torben Gentz (MICADAS, AWI Bremerhaven) are acknowledged for conducting the radiocarbon dating. Lennard Klingforth supported the data acquisition (shells/ImageJ). The BSH is thanked for providing modelled current data. This study was funded by the AWI Section Coastal Ecology and the project RESTORE (FKZ 3516892001).

**References**

[1] OSPAR. Background document for *Ostrea edulis* and *Ostrea edulis* beds. London: OSPAR Commission; 2009. 21 p.

[2] Bromley C, McGonigle C, Ashton EC, Roberts D. Bad moves: Pros and cons of moving oysters – A case study of global translocations of *Ostrea edulis* Linnaeus, 1758 (Mollusca: Bivalvia). Ocean Coast. Manage. 2016; 122:103-115.

[3] Thorngren L, Bergström P, Holthuis TD, Lindegarth M. Assessment of the population of Ostrea edulis in Sweden: A marginal population of significance?. Ecol. Evol. 2019; 9(24):13877-13888.

[4] Hayer S, Bick A, Brandt A, Ewers-Saucedo C, Fiege D, Füting S, Krause-Kyora B, Michalik P, Reinicke G-B, Brandis D. Coming and going – Historical distributions of the European oyster Ostrea edulis Linnaeus, 1758 and the introduced slipper limpet Crepidula fornicata Linnaeus, 1758 in the North Sea. PLOS ONE. 2019;14(10): e0224249.

[5] Caspers H. Die Lebensgemeinschaft der Helgoländer Austernbank. Helgoland Wiss. Meer. 1950; 3(1):119.

[6] Boos K, Buchholz C, Buchholz F, Gutow L. Bericht über die Zusammensetzung des Helgoländer Makrozoobenthos im Vergleich historischer und aktueller Quellen: Klassifizierungsvorschlag nach der WRRL und Empfehlungen zum Monitoring. Flintbek: Landesamt für Natur und Umwelt (LANU) des Landes Schleswig-Holstein; 2004. 94 p.

[7] Smyth D, Roberts D. The European oyster (Ostrea edulis) and its epibiotic succession. Hydrobiologia. 2010; 655:25-36.

[8] Kamermans P, Walles B, Kraan M, van Duren LA, Kleissen F, van der Have TM, Smaal AC, Poelman M. Offshore wind farms as potential locations for flat oyster (Ostrea edulis) restoration in the Dutch North Sea. Sustainability. 2018; 10:3942

[9] Pogoda B, Brown J, Hancock B, Preston J, Pouvreau S, Kamermans P, Sanderson W, von Nordheim H. The Native Oyster Restoration Alliance (NORA) and the Berlin Oyster Recommendation: bringing back a key ecosystem engineer by developing and supporting best practice in Europe. Aquat. Living Resour. 2019; 32:13.

[10] Korringa P. The decline of natural oyster beds. Basteria. 1946; 10(3/4):36-41.

[11] OSPAR. Ostrea edulis, Flat Oyster. In: OSPAR Commission, editor. Case Reports for the OSPAR List of threatened and/or declining species and habitats. London: OSPAR Commission; 2008. pp. 243-246.

[12] Thurstan RH, Hawkins JP, Raby L, Roberts CM. Oyster (Ostrea edulis) extirpation and ecosystem transformation in the Firth of Forth, Scotland. J. Nat. Conserv. 2013; 21(5):253-261.

[13] Pogoda B. Current Status of European Oyster Decline and Restoration in Germany. Humanities. 2019; 8(1):9

[14] Beck, MW, Brumbaugh RD, Airoldi L, Carranza A, Coen LD, Crawford C, Defeo O, Edgar GJ, Hancock B, Kay MC, Lenihan HS, Luckenbach MW, Toropova CL, Zhang G, Guo X. Oyster reefs at risk and recommendations for conservation, restoration, and management. Bioscience. 2011; 61(2):107-116.

[15] Gercken J, Schmidt A. Current status of the European Oyster (Ostrea edulis) and possibilities for restoration in the German North Sea. BfN-Skripten 2014. Bonn: Bundesamt für Naturschutz BfN, 107p.

[16] Smaal AC, Kamermans P, Van der Have TM, Engelsma MY, Sas H. Feasibility of Flat Oyster (Ostrea edulis L.) restoration in the Dutch part of the North Sea. Ijmuiden: Institute for Marine Resources & Ecosystem Studies, Report No. C028/15; 2015. 58 p.

[17] Dankers N, Herlyn M, Kristensen P, Michaelis H, Millat G, Nehls G, Ruth M. Blue mussels and blue mussel beds in the littoral. In: De Jong F, Bakker J, van Berkel C, Dahl K, Dankers N, Gätje C, Marencic H, Potel P, editors. Wadden Sea quality status report. Wilhelmhaven: Common Wadden Sea Secretariat, 1999. pp. 141-145.

[18] Kerckhof F, Coolen JW, Rumes B, Degraer S. Recent findings of wild European flat oysters Ostrea edulis (Linnaeus, 1758) in Belgian and Dutch offshore waters: new perspectives for offshore oyster reef restoration in the southern North Sea. Belg. J. Zool. 2018;148(1):13-24.

[19] Pogoda B, Merk V, Colsoul B, Hausen T, Peter C, Pesch R, Kramer M, Jaklin S, Holler P, Bartholomä A, Michaelis R, Prinz K. Site selection for biogenic reef restoration in offshore environments: The Natura 2000 area Borkum Reef Ground as a case study for native oyster restoration. Aquat. Conserv. 2020, accepted.

[20] Olsen OT. The Piscatorial Atlas of the North Sea and St. George's Channels. London: Taylor and Francis; 1883.

[21] Fariñas-Franco JM, Pearce B, Mair JM, Harries DB, MacPherson RC, Porter JS, Reimer P, Sanderson WG. Missing native oyster (Ostrea edulis L.) beds in a European Marine Protected Area: Should there be widespread restorative management?. Biol. Conserv. 2018; 221:293-311.

[22] Anonymous. Die Nordsee-Austernfischerei. Fischerbote. 1913; 5:288-289.

[23] Lotze HK. Radical changes in the Wadden Sea fauna and flora over the last 2,000 years. Helgoland Mar. Res. 2005; 59(1):71-83.

[24] Hasselmann ZW. Versuch einer Beschreibung der Insel Helgoland. In: Niemann ACH, editor. Schleswig-Holsteinische Provinzialberichte. Leipzig: Hammerich; 1792. pp. 1-21.

[25] Schnakenbeck W. Die Nordseefischerei. In: Lübbert H, Ehrenbaum E, editors. Handbuch der Seefischerei Nord-Europas, 5(1), Stuttgart: Schweizerbart’sche Verlagsbuchhandlung. 1928.

[26] Möbius KA. Die Austern und die Austernwirtschaft. Berlin: Hepel und Parey Verlag von Wiegandt; 1877. 126p.

[27] Seaman MN, Ruth M. The molluscan fisheries of Germany. In: MacKenzie Jr CL, Burrell Jr VG, Rosenfield A, Hobart WL, editors. The History, Present Condition, and Future of the Molluscan Fisheries of North and Central America and Europe, Volume 3, Europe. US Dep. Commer., NOAA Tech. Rep. NMFS 129. Seattle (WA): U.S. Department of Commerce; 1997. pp.57-84.

[28] zu Ermgassen PSE, Hancock B, DeAngelis B, Greene J, Schuster E, Spalding M, Brumbaugh R. Setting objectives for oyster habitat restoration using ecosystem services: a manager’s guide. Arlington (VA): TNC; 2016. 76 p.

[29] Laing I, Walker P, Areal F. Return of the native – is European oyster (Ostrea edulis) stock restoration in the UK feasible? Aquat. Living Resourc. 2006; 19:283-287.

[30] Michaelis R, Hass HC, Mielck F, Papenmeier S, Sander L, Ebbe B, Gutow L, Wiltshire KH. Hard-substrate habitats in the German Bight (South-Eastern North Sea) observed using drift videos. J. Sea Res. 2019; 144:78-84.

[31] Janssen F, Schrum C, Backhaus JO. A climatological data set of temperature and salinity for the Baltic Sea and the North Sea. German J. Hydrography 1999; S9:245 p.

[32] Quante M, Colijn F, Bakker JP, Härdtle W, Heinrich H, Lefebvre C, Nöhren I, Olesen JE, Pohlmann T, Sterr H, Sündermann J, Tölle MH. Introduction to the Assessment: Characteristics of the Region. In: Quante M, Colijn F, editors. North Sea Region Climate Change Assessment. Cham: Springer, 2016. pp. 1-52.

[33] Eisma D. Supply and deposition of suspended mater in the North Sea. Spec. Publs. Int. Ass. Sediment. 1981; 5:415-428.

[34] Irion G, Wunderlich F, Schwedhelm E. Transport of clay minerals and anthropogenic compounds into the German Bight and the provenance of fine-grained sediments SE of Helgoland. J. Geol. Soc. London. 1987; 144:153-160.

[35] Lee A, Ramster J. The hydrography of the North Sea. A review of our knowledge in relation to pollution problems. Helgoland Wiss. Meer. 1968; 17(1):44.

[36] OSPAR. Quality Status Report 2000, Region II – Greater North Sea. London: OSPAR Commission; 2000. 136 p.

[37] Siegismund F, Schrum C. Decadal changes in the wind forcing over the North Sea. Clim. Res. 2001; 18(1-2):39-45.

[38] Staneva J, Stanev EV, Wolff J-O, Badewien TH, Reuter R, Flemming B, Bartholomä A, Bolding K. Hydrodynamics and sediment dynamics in the German Bight. A focus on observations and numerical modelling in the East Frisian Wadden Sea. Cont. Shelf Res. 2009; 29:302–319.

[39] Otto L, Zimmerman JTE, Furnes GK, Mork M, Saetre R, Becker G. Review of the physical oceanography oft he North Sea. Neth. J. Sea Res. 1990; 26 (2-4):161-238.

[40] Spaeth C. Zur Geologie der Insel Helgoland. Die Küste. 1990; 49:1-32.

[41] Hass HC, Mielck F, Fiorentino D, Papenmeier S, Holler P, Bartholomä A. Seafloor monitoring west of Helgoland (German Bight, North Sea) using the acoustic ground discrimination system RoxAnn. Geo-Mar. Lett. 2017; 37(2):125-136.

[42] BSH, 2018. Meeresboden DGM. http://www.geoseaportal.de/ (accessed 08. May 2018).

[43] Valerius J, Kösters F, Zeiler M. Erfassung von Sandverteilungsmustern zur großräumigen Analyse der Sedimentdynamik auf dem Schelf der Deutschen Bucht. Die Küste; 2015;83:39-63

[44] IOC, IHO, BODC. Centenary Edition of the GEBCO Digital Atlas. Liverpool: British oceanographic data centre. 2003.

[45] Dick S, Kleine E, Müller-Navarra SH, Klein H, Komo H. The operational circulation model of BSH (BSHcmod)–model description and validation. Berichte des BSH. 2001; 29:1-48.

[46] Rueden CT, Schindelin J, Hiner MC. ImageJ2: ImageJ for the next generation of scientific image data. BMC Bioinformatics. 2017; 18(1):529.

[47] Bronk Ramsey C. Bayesian analysis of radiocarbon dates. Radiocarbon 2009;51(1):337-360.

[48] Reimer PJ, Bard E, Bayliss A, Beck JW, Blackwell PG, Bronk Ramsey C, Grootes PM, Guilderson TP, Haflidason H, Hajdas I, Hattz C, Heaton TJ, Hoffmann DL, Hogg AG, Hughen KA, Kaiser KF, Kromer B, Manning SW, Niu M, Reimer RW, Richards DA, Scott EM, Southon JR, Staff RA, Turney CSM, van der Plicht J. IntCal13 and Marine13 Radiocarbon Age Calibration Curves 0-50,000 Years cal BP. Radiocarbon. 2013; 55(4):1869-1887.

[49] Heier-Nielsen S, Heinemeier J, Nielsen HL, Rud N. Recent reservoir ages for Danish fjords and marine waters. Radiocarbon. 1995; 37(3):875-882.

[50] Perry F, Jackson A, Garrard SL. Ostrea edulis Native oyster. In: Tyler-Walters H, Hiscock K, editors. Marine Life Information Network: Biology and Sensitivity Key Information Reviews. Plymouth: Marine Biological Association of the United Kingdom; 2017. Available from: https://www.marlin.ac.uk/species/detail/1146 (last accessed:11.03.2020).

[51] Hoeksema BW. Excavation patterns and spiculae dimensions of the boring sponge Cliona celata from the SW Netherlands. Senck. Marit. 1983; 15:55-85.

[52] Harding JM, Mann R, Southworth MJ. Shell length-at-age relationships in James River, Virginia, oysters (Crassostrea virginica) collected four centuries apart. J. Shellfish Res. 2008; 27(5):1109-1116.

[53] Mann R, Harding JM, Southworth MJ. Reconstructing pre-colonial oyster demographics in the Chesapeake Bay, USA. Estuar. Coast. Shelf S. 2009; 85(2):217-222.

[54] Helmer L, Farrell P, Hendy I, Harding S, Robertson M, Preston J. Active management is required to turn the tide for depleted Ostrea edulis stocks from the effects of overfishing, disease and invasive species. PeerJ. 2019; 7:e6431.

[55] Warburton F. The effects of boring sponges on oysters. Fish. Res. Bd. Can. Progr. Rep. Atl. Coast. Stns. 1958; 68:3-8.

[56] Hartman WD. Natural history of the marine sponges of southern New England. New Haven (CT): Peabody Museum of Natural History; 1958. 155 p.

[57] Hansen JC, 1970. Commensal activity as a function of age in two species of California abalones (Mollusca: Gastropoda). Veliger. 1970; 13:90-94

[58] Carroll JM, O´Shaughnessy KA, Diedrich GA, Finelli CM. Are oysters being bored to death? Influence of Cliona celata on Crassostrea virginica condition, growth and survival. Dis. Aquat. Organ. 2015; 117:31-44.

[59] Hargis Jr WJ. The evolution of the Chesapeake oyster reef system during the Holocene Epoch. In: Luckenbach M, Mann R, Wesson JA, editors. Oyster reef habitat restoration: a synopsis of approaches. Gloucester Point (VA): Virginia Institute of Marine Science; 1999. pp. 5-24.

[60] Lenihan HS. Physical-biological coupling on oyster reefs: How habitat structure influences individual performance. Ecol. Monogr. 1999; 69(3):251–275.

[61] Vink A, Steffen H, Reinhardt L, Kaufmann G. Holocene relative sea-level change, isostatic subsidence and the radial viscosity structure of the mantle of northwest Europe (Belgium, the Netherlands, Germany, southern North Sea). Quaternary Sci. Rev. 2007; 26(25-28):3249-3275.

[62] Andersen, SH. Shell middens (“Køkkenmøddinger”) in Danish prehistory as a reflection of the marine environment. In: Milner N, Craig OE, Bailey GN, editors. Shell middens in Atlantic Europe. Oxford: Oxbow Books Limited; 2007. pp. 31-45.

[63] Bennike O, Nørregaard-Petersen N, Jensen JB, Andresen KJ, Seidenkrantz MS. Development of the western Limfjord, Denmark, after the last deglaciation: a review with new data. Bull. Geol. Soc. Denmark. 2019 ;67(1).

[64] Hebbeln D, Scheurle C, Lamy F. Depositional history of the Helgoland mud area, German Bight, North Sea. Geo-Mar. Lett. 2003; 23(2):81-90.

[65] Reineck HE, Gutmann WF, Hertweck G. Das Schlickgebiet südlich Helgoland als Beispiel rezenter Schelfablagerungen. Senck. Lethaea. 1967; 48:219-275.

[66] McCave IN. Deposition of fine-grained suspended sediment from tidal currents. J. Geophys. Res. 1970; 75(21):4151-4159.

[67] Kaplan JO, Krumhardt, KM, Zimmermann N. The prehistoric and preindustrial deforestation of Europe. Quaternary Sci. Rev. 2009; 28:3016–3034.

[68] Hoffmann T, Lang A, Dikau R. Holocene river activity: analysing 14C-dated fluvial and colluvial sediments from Germany. Quaternary Sci. Rev. 2008; 27(21-22):2031-2040.

[69] Lang A, Bork H-R, Mäckel R, Preston N, Wunderlich J, Dikau R. Changes in sediment flux and storage within a fluvial system: some examples from the Rhine catchment. Hydrol. Process. 2003; 17:3321-3334.

[70] Schneeweiss J, Schatz T. The impact of landscape change on the significance of political centres along the lower Elbe River in the 10th century AD. Quatern. Int. 2014; 324:20-33.

[71] Becker GA, Dick S, Dippner JW. 1992. Hydrography of the German bight. Mar. Ecol. Prog. Ser. 1992; 91:9-18.

[72] Orme LC, Davies SJ, Duller GAT. Reconstructed centennial variability of Late Holocene storminess from Cors Fochno, Wales, UK. J. Quaternary Sci. 2015; 30(5):478-488.

[73] Charbotte SM, Bell RE, Ryan WBF, McHugh C, Slagle A, Nitsche F, Rubenstone J. Environmental change and oyster colonization within the Hudson River estuary linked to Holocene climate. Geo-Mar. Lett. 2004; 24:212-224.

[74] Lewis JP, Ryves DB, Rasmussen P, Olsen J, Knudsen KL, Andersen SH, Weckström K, Clarke AL, Andrén E, Juggins S. The shellfish enigma across the Mesolithic-Neolithic transition in southern Scandinavia. Quaternary Sci. Rev. 2016; 151:315-320.

[75] Rodriguez-Perez A, James M, Donnan DW, Henry TB, Møller LF, Sanderson WG. Conservation and restoration of a keystone species: Understanding the settlement preferences of the European oyster (Ostrea edulis). Mar. Poll. Bull. 2019; 138:312-321.

[76] Pollack JB, Cleveland A, Palmer TA, Reisinger AS, Montagna PA. A Restoration Suitability Index Model for the Eastern Oyster (Crassostrea virginica) in the Mission-Aransas Estuary, TX, USA. PLOS ONE. 2012; 7(7):e40839.
